# A Comparative Analysis of the Anti-Tumor Activity of Sixteen Polysaccharide Fractions from Three Large Brown Seaweed, *Sargassum horneri*, *Scytosiphon lomentaria,* and *Undaria pinnatifida*

**DOI:** 10.3390/md22070316

**Published:** 2024-07-16

**Authors:** Lin Song, Yunze Niu, Ran Chen, Hao Ju, Zijian Liu, Bida Zhang, Wancui Xie, Yi Gao

**Affiliations:** 1 Shandong Provincial Key Laboratory of Biochemical Engineering, College of Marine Science and Biological Engineering, Qingdao University of Science and Technology, Qingdao 266042, China; 03231@qust.edu.cn (L.S.); xiewancui@163.com (W.X.); 2 Wuqiong Food Co., Ltd., Raoping 515726, China; 3 College of Life Sciences, Qingdao Agricultural University, Qingdao 266109, China; niuyunze1997@163.com (Y.N.); cr1164365956@163.com (R.C.); jh19981110@163.com (H.J.); 15764200096@163.com (Z.L.); 4 Changdao Aihua Seaweed Food Co., Ltd., Yantai 265800, China; 5 College of Marine Science and Engineering, Qingdao Agricultural University, Qingdao 266237, China

**Keywords:** brown seaweed, polysaccharide, sixteen fractions, anti-tumor activity, comparative analysis

## Abstract

Searching for natural products with anti-tumor activity is an important aspect of cancer research. Seaweed polysaccharides from brown seaweed have shown promising anti-tumor activity; however, their structure, composition, and biological activity vary considerably, depending on many factors. In this study, 16 polysaccharide fractions were extracted and purified from three large brown seaweed species (*Sargassum horneri*, *Scytosiphon lomentaria,* and *Undaria pinnatifida*). The chemical composition analysis revealed that the polysaccharide fractions have varying molecular weights ranging from 8.889 to 729.67 kDa, and sulfate contents ranging from 0.50% to 10.77%. Additionally, they exhibit different monosaccharide compositions and secondary structures. Subsequently, their anti-tumor activity was compared against five tumor cell lines (A549, B16, HeLa, HepG_2,_ and SH-SY5Y). The results showed that different fractions exhibited distinct anti-tumor properties against tumor cells. Flow cytometry and cytoplasmic fluorescence staining (Hoechst/AO staining) further confirmed that these effective fractions significantly induce tumor cell apoptosis without cytotoxicity. qRT-RCR results demonstrated that the polysaccharide fractions up-regulated the expression of *Caspase-3*, *Caspase-8*, *Caspase-9*, and *Bax* while down-regulating the expression of *Bcl-2* and *CDK-2*. This study comprehensively compared the anti-tumor activity of polysaccharide fractions from large brown seaweed, providing valuable insights into the potent combinations of brown seaweed polysaccharides as anti-tumor agents.

## 1. Introduction

Cancer is one of the most serious diseases leading to death in the world, especially in Asia, characterized by the growth of malignant tumor cells [1]. According to the GLOBOCAN report, the global cancer burden in 2022 showed approximately 20 million new cases of cancer and 9.7 million deaths worldwide, with an estimated 49.2% of cancer cases and 56.1% of deaths occurring in Asia [2]. Natural seaweed polysaccharides, including alginates, laminarans, fucoidans, carrageenan, and agar, are considered potential candidates against cancer due to their strong anti-tumor activities and minimal side effects [3,4]. Seaweed polysaccharides have been proven to promote anti-tumor effects and can be used as adjuvant therapies for cancer [4]. Various bioactive substances with anti-tumor activity have been identified in seaweed polysaccharides, such as sulfated polysaccharides and fucoidans [5,6].

Brown seaweed, the second-largest class of seaweed, contains a large amount of bioactive polysaccharides, primarily alginate, fucoidan, and laminarin, which account for 40–80% of the dry seaweed biomass, indicating that brown seaweed is a rich source of these functional polysaccharides [7,8]. As a traditional part of Oriental diets and herbal medicine, brown seaweed has a long history in Asian countries and is more easily accepted by the local people [9]. In recent years, there has been increasing attention paid to the structures and potential biological activities of brown seaweed polysaccharides [10,11]. Studies have shown that polysaccharides extracted from brown seaweed contain high levels of sulfates and polyphenols, and they exhibit higher antioxidant activity compared to green and red seaweed [12]. Research has also highlighted the anti-tumor properties of polysaccharides extracted from brown seaweed, indicating the potential use of brown seaweed as functional ingredients with therapeutic characteristics [13,14]. In our previous study, representatives from three major classes of seaweed (red seaweed, brown seaweed, and green seaweed) were selected to compare the anti-tumor activity of their polysaccharides, and the results showed that brown seaweed polysaccharides exhibited stronger anti-tumor activity. The high anti-tumor activity of brown seaweed is attributed to its unique chemical structure, such as sulfate content, molecular weight, and monosaccharide composition [15]. 

Research has shown that seaweed polysaccharides exhibit unique properties in terms of structure, composition, and biological activity, which depend on factors such as the source of seaweed and extraction methods [8,11,16]. Similarly, the biological activity of seaweed polysaccharides against cancer cells varies, and they exhibit specificity in their anti-tumor activity against different tumor cells [6,17,18,19]. For example, when extracting and purifying polysaccharides from *Sargassum pallidum* to study their anti-tumor activity, Ye et al. (2008) identified seven different polysaccharide fractions. Among them, the low molecular weight fractions SP-3-1 and SP-3-2 showed higher anti-tumor activity against the HepG_2_, A549, and MGC-803 cells than the high molecular weight fractions [20]. Li et al. (2017) isolated and purified four fractions from *S. pallidum* and studied their activity against HepG_2_ cells and the results revealed that SP-P2 (medium molecular weight) exhibited the strongest anti-tumor activity against HepG_2_ cells, followed by SP-P4, SP-P1, and SP-P3 [21]. Gao et al. isolated and purified five polysaccharide fractions (SPP-0.3, SPP-0.5, SPP-0.7, SPP-1, and SPP-2) from *S. pallidum* in 2021. The biological activity analysis showed that all of these five fractions possessed anti-tumor activity against three cancer cells (A549, HepG_2_, and B16), with SPP-0.7 (medium molecular weight) being identified as the most active fraction [22]. It can be observed that even for the same type of seaweed, the anti-tumor effects of polysaccharide fractions obtained under different experimental conditions or extraction methods may vary for different tumor cells [8]. Therefore, it is necessary to compare the anti-tumor activities of seaweed polysaccharides from different sources against different tumor cells as well as their structure–function relationship in order to identify the most effective anti-tumor activity components for different tumor cells. 

*Sargassum horneri*, *Scytosiphon lomentaria*, and *Undaria pinnatifida* are three common large brown seaweed in Asia and are commonly consumed by the local population. Current research on these three seaweed indicates that their polysaccharides possess various biological activities such as antiviral, hypoglycemic, hypolipidemic, antioxidant, and immunomodulatory effects [23,24,25,26,27] (Table 1). However, there is limited research on the anti-tumor activity of polysaccharides from these three seaweed, with only a few reported cases; for example, Shao et al. demonstrated the anti-tumor activity of *S. horneri* polysaccharides against human colon carcinoma (DLD) cells through flow cytometry and qRT-PCR. It is speculated that the polysaccharides may inhibit DLD cell proliferation and induce DLD cell apoptosis by affecting the expression of apoptosis-related genes such as Bcl-2 and Bax [28,29]. Rasin et al. used recombinant fucoidanase to hydrolyze fucoidan of *S. horneri* and separated the high molecular weight fractions using anion-exchange chromatography. They obtained three sulfated polysaccharide fractions and found that the F3 fraction exhibited the strongest anti-tumor (human colon carcinoma cancer) activity [23]. Fucoidan from *U. pinnatifida* was evaluated over different melanoma cells lines, and the results demonstrated that the addition of fucoidan to lapatinib inhibited melanoma cells’ viability in 85% of cell lines [30]. Research on the anti-tumor activity of polysaccharides from *S. lomentaria* is still lacking. Although previous studies have reported the anti-tumor effect of these seaweed polysaccharides, most of them focused on individual polysaccharides targeting specific tumor cell lines (Table 1), and research on the anti-tumor activity of their fractions is scarce. Furthermore, there is insufficient research on the anti-tumor activity of purified polysaccharide fractions from these three seaweed and a lack of comparative studies on their anti-tumor activities.

Therefore, this study aimed to compare the anti-tumor effects of three brown seaweed polysaccharides, *S. horneri*, *S. lomentaria*, and *U. pinnatifida*. For this purpose, the polysaccharides were extracted and purified, and the structural differences between fractions were compared. The anti-tumor activity of the polysaccharide fractions from the three brown seaweed was evaluated, and a further investigation into their anti-tumor mechanisms was conducted. The results of this study will provide support for obtaining seaweed polysaccharides with highly efficient anti-tumor activity in the future. 

## 2. Results

### 2.1. Extraction, Purification, and Characterization of 16 Polysaccharide Fractions from Three Types of Brown Seaweed

Initially, three crude polysaccharides were extracted from *S. horneri*, *S. lomentaria,* and *U. pinnatifida* using hot water extraction, ethanol precipitation, dialysis, and freeze-drying. The polysaccharides were then deproteinized and purified through chromatography using a DEAE-Sepharose fast-flow column. The eluted fractions were collected and dialyzed, and free proteins were eliminated. As a result, a total of 16 polysaccharide fractions were purified from the three types of seaweed polysaccharides. Specifically, seven fractions named SHP-0.3, SHP-0.5, SHP-0.7, SHP-1.0, SHP-1.3, SHP-1.5, and SHP-1.7 were purified from the *S. horneri* polysaccharide based on their elution salinity. Likewise, five fractions named SLP-0.3, SLP-0.5, SLP-0.7, SLP-1.3, and SLP-1.5 were purified from the *S. lomentaria* polysaccharide. Additionally, four fractions named UPP-0.3, UPP-0.5, UPP-1.0, and UPP-1.3 were obtained from the *U. pinnatifida* polysaccharide (Figure S1). 

The sulphate content, total sugar content, monosaccharide composition, and molecular weight of 16 types of fractionated polysaccharide samples were determined (Table 2, Table 3 and Table 4). Sulphate content determination showed that all 16 polysaccharide samples contained sulphate groups. The top six fractions with the highest sulphate content were SHP-1.7 (10.77%), SHP-1.5 (8.63%), SHP-0.3 (7.62%), SHP-0.7 (6.91%), SHP-0.5, and SLP-0.7 (6.49%). Total sugar content determination showed that the top five samples with the highest total sugar content were UPP-0.3 (90%), SLP-0.7 (75%), SLP-1.3 (60%), UPP-1.0 (42%), and SHP-1.7 (37%). The monosaccharide composition of each polysaccharide was determined, and the results show that the monosaccharide composition of all of the fractionated polysaccharide samples was different. The majority of polysaccharide samples were mainly composed of mannose, galacturonic acid, and xylose. The highest molecular weight of SHP, SLP, and UPP fractions was SHP-1.7 (729.67 kDa), SLP-1.5 (646.83 kDa), and UPP-1.3 (444.96 kDa), respectively. These results indicate that there are significant differences in the chemical compositions of these fractionated polysaccharide samples.

### 2.2. Fourier Transform Infrared (FT-IR) Spectroscopy Analysis of 16 Polysaccharide Fractions

**Figure 1:**
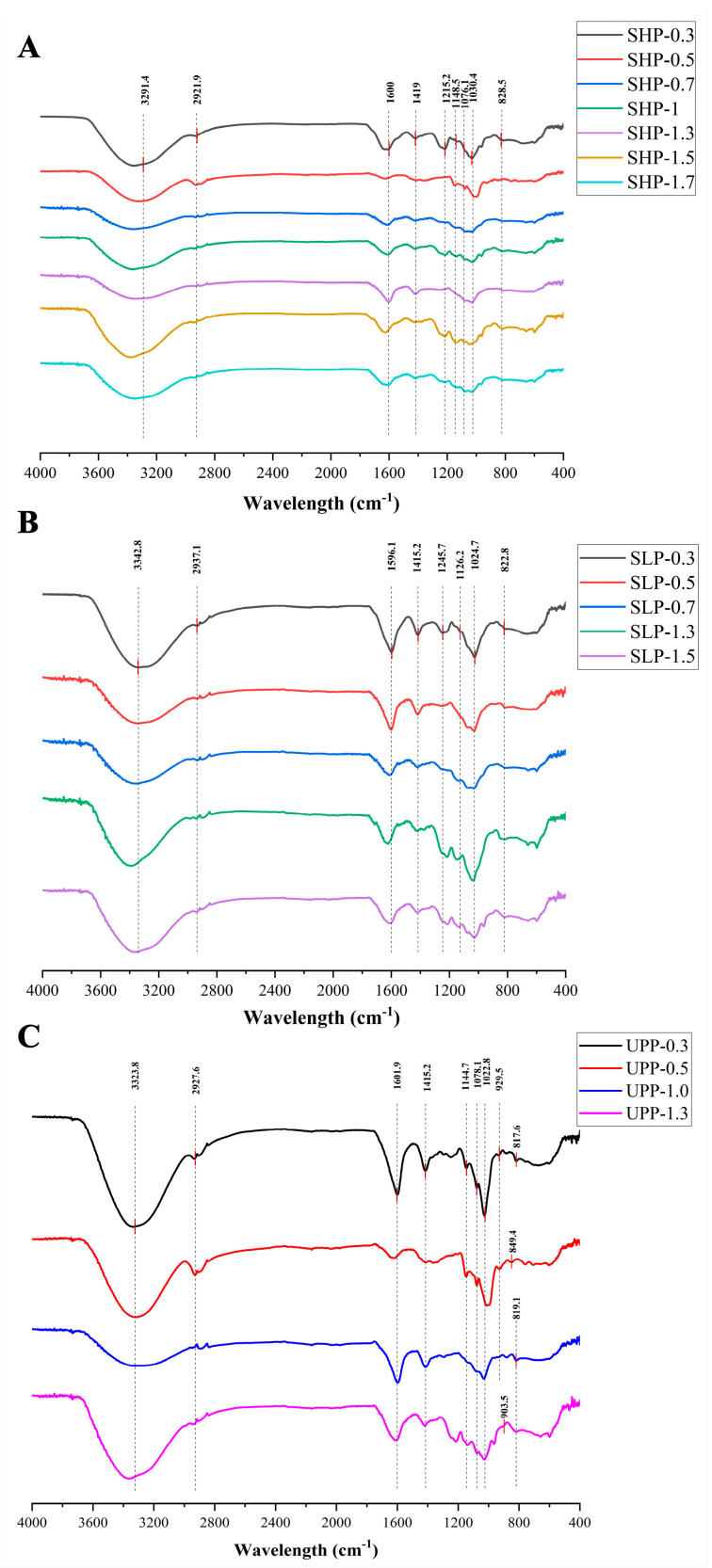
Fourier transform infrared (FT-IR) spectra of polysaccharide fractions from three types of seaweed. (**A**) *Sargassum horneri* polysaccharide (SHP) fractions, (**B**) *Scytosiphon lomentaria* polysaccharide (SLP) fractions, and (**C**) *Undaria pinnatifida* polysaccharide (UPP) fractions.

FT-IR spectroscopy was employed to gain further insights into the structure of polysaccharide fractions. The findings revealed common absorption peaks shared by all polysaccharide fractions (Figure 1). Specifically, the O-H stretching vibrations (3400–3200 cm^−1^) and C–H stretching vibrations (2920–2940 cm^−1^) were identified as the characteristic absorption peaks of polysaccharides [47,48]. Additionally, the peaks observed around 1240–1260 cm^−1^ and 810–840 cm^−1^ were attributed to the sulfate ester groups in the polysaccharide fractions, corresponding to the S=O stretching vibration and C-O-S bending vibration, respectively [49,50,51].

For the SHP fractions, the peak near 1600 cm^−1^ was considered the characteristic absorption peak of alkene (-C=C-) [51], while the peak around 1419 cm^−1^ corresponded to the bending vibration of the C-H bond [52]. In the case of SHP-0.3, SHP-1, SHP-1.5, and SHP-1.7 fractions, the peak observed around 1215.2 cm^−1^ was attributed to the symmetric stretching vibration of C-H [53]. The absorption bands ranging from 1000 cm^−1^ to 1200 cm^−1^ (1148.5, 1076.1, and 1030.4 cm^−1^) suggested the presence of C-O-C and C-O-H groups in the SHP fractions, which are characteristic of pyranose [54]. Furthermore, the signal near 828.5 cm^−1^ indicated the presence of an α-type glycosidic linkage in SHP (Figure 1A) [43].

For the SLP fractions, the peak near 1596 cm^−1^ was considered characteristic of alkene (-C=C-) [51], while the peak around 1415.2 cm^−1^ represented the bending vibration of the C-H bond [52]. The peak around 1215.2 cm-1 in SLP-0.3, SLP-1.3, and SLP-1.5 fractions was attributed to the symmetric stretching vibration of C-H [53]. Absorption peaks in the range from 1000 cm^−1^ to 1200 cm^−1^ (1126.2 and 1024.7 cm^−1^) suggested the presence of C-O-C and C-O-H groups in SLP fractions, characteristic of pyranose [54]. Additionally, the signal near 822.8 cm^−1^ suggested the presence of an α-type glycosidic linkage in SLP (Figure 1B) [43].

For the UPP fractions, the peak near 1601.9 cm^−1^ was considered characteristic of alkene (-C=C-). The peak around 1415.2 cm^−1^ was the bending vibration of the C-H bond [52]. The absorption bands around 1144.7, 1078.1, and 1022.8 cm^−1^ indicated the presence of C-O-C and C-O-H groups in UPP, characteristic of pyranose [54]. The peak close to 920 cm^−1^ was associated with a β-type glycosidic linkage, and there were also α-type glycosidic linkages (817.6, 849.4, and 819.1 cm^−1^) (Figure 1C) [43].

### 2.3. Triple-Helix Conformation

**Figure 2:**
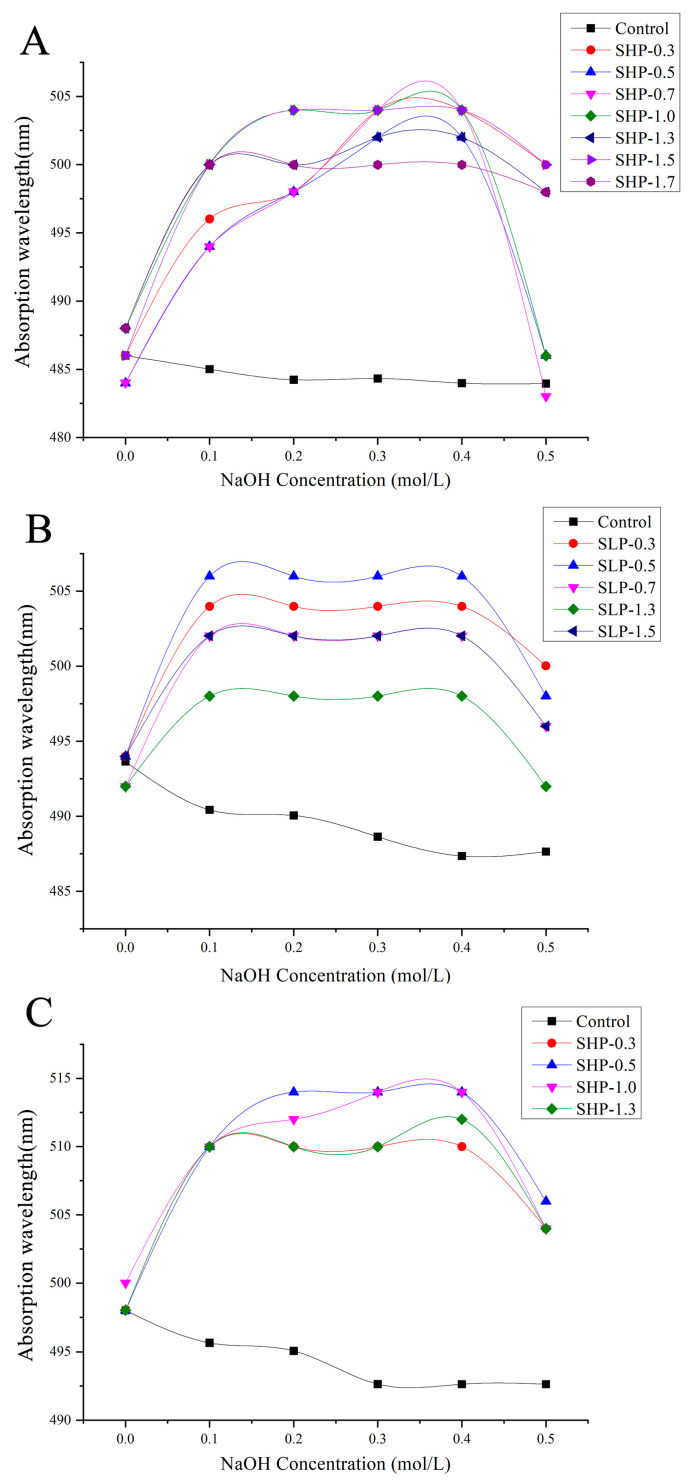
The triple-helix conformation analysis of polysaccharide fractions from three types of seaweed. (**A**) *S. horneri* polysaccharide (SHP) fractions, (**B**) *S. lomentaria* polysaccharide (SLP) fractions, and (**C**) *U. pinnatifida* polysaccharide (UPP) fractions.

Congo red can form a complex with polysaccharides in a triple-helix structure, resulting in a redshift in the maximum absorption wavelength (λ_max_). As shown in Figure 2, the λ_max_ of these polysaccharide fractions with Congo red in sodium hydroxide solutions of various concentrations was observed to change. Compared to the control group, a redshift in λ_max_ was observed in all solutions. These results indicate that all fractions possess a triple-helix structure. 

### 2.4. Comparison of Anti-Tumor Activities of 16 Polysaccharide Fractions

**Figure 3:**
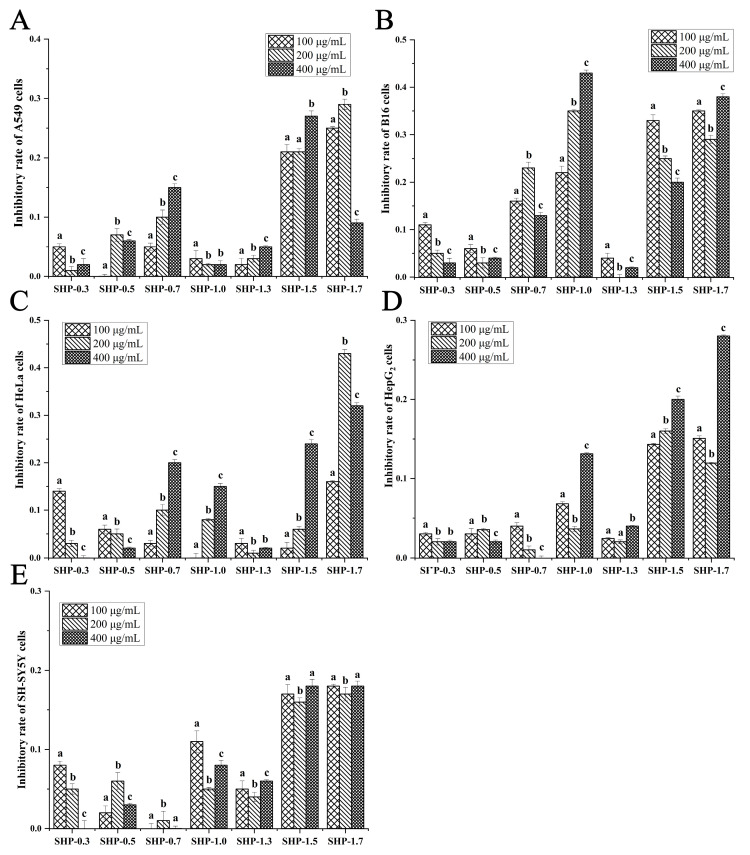
The inhibitory effects of seven fractions of the *S. horneri* polysaccharide (SHP) at different concentrations (100, 200, and 400 μg/mL) on the proliferation of (**A**) A549, (**B**) B16, (**C**) HeLa, (**D**) HepG_2_, and (**E**) SH-SY5Y cells (mean  ±  standard deviation). The letters a–c indicate significant differences (*p*  <  0.05) in the inhibitory rate of each fraction.

**Figure 4:**
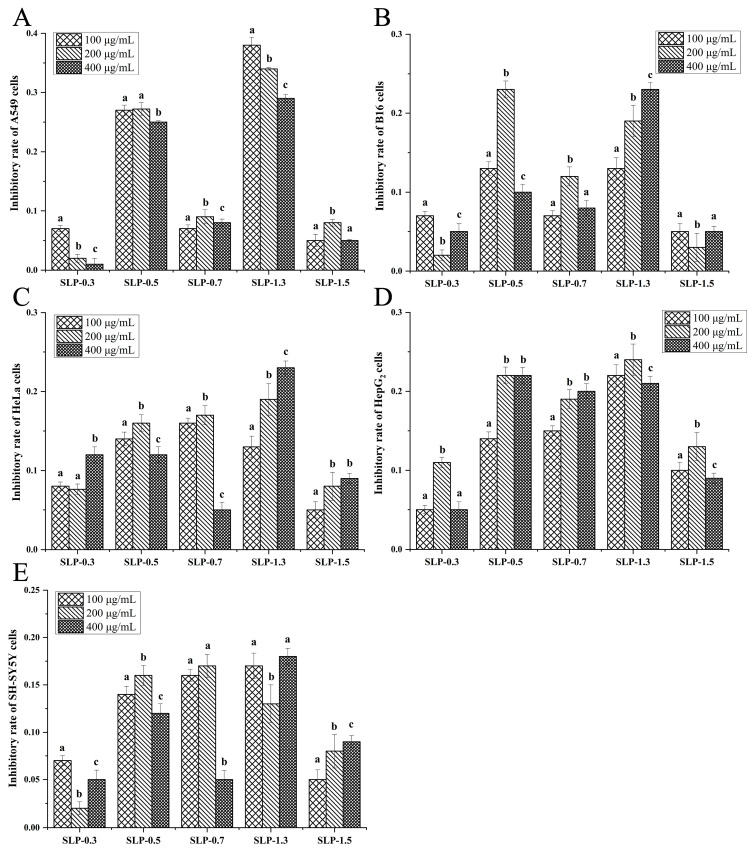
The inhibitory effects of five fractions of the *S. lomentaria* polysaccharide (SLP) at different concentrations (100, 200, and 400 μg/mL) on the proliferation of (**A**) A549, (**B**) B16, (**C**) HeLa, (**D**) HepG_2_, and (**E**) SH-SY5Y cells (mean  ±  SD). The letters a–c indicate significant differences (*p*  <  0.05) in the inhibitory rate of each fraction.

**Figure 5:**
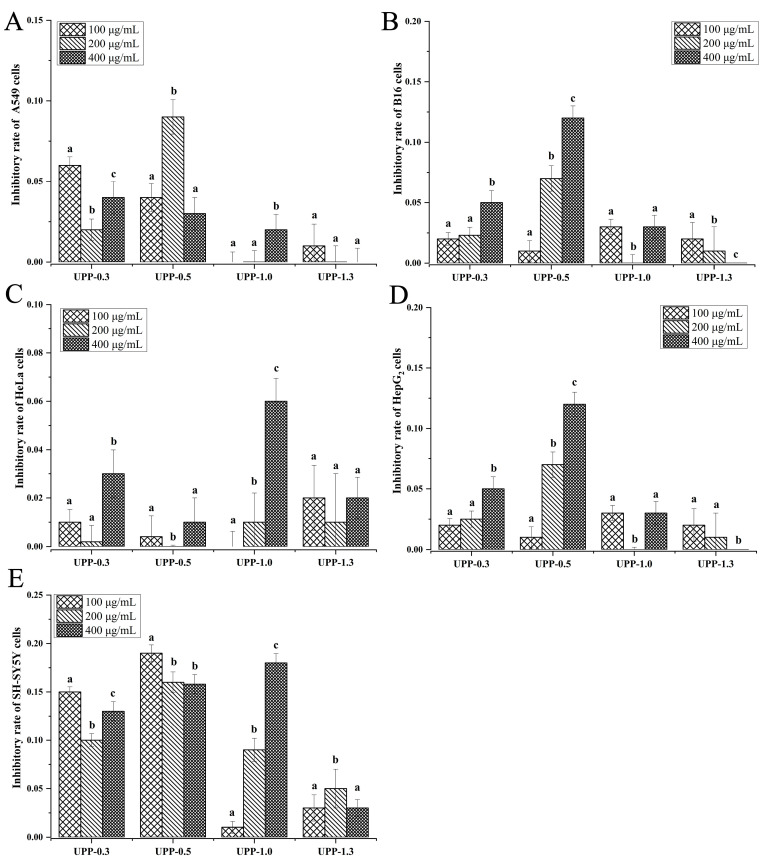
The inhibitory effects of four fractions of the *U. pinnatifida* polysaccharide (UPP) at different concentrations (100, 200, and 400 μg/mL) on the proliferation of (**A**) A549, (**B**) B16, (**C**) HeLa, (**D**) HepG_2_, and (**E**) SH-SY5Y cells (mean  ±  SD). The letters a–c indicate significant differences (*p*  <  0.05) in the inhibitory rate of each fraction.

To evaluate the anti-tumor activity of 16 polysaccharide fractions on different tumor cells, the growth inhibitory effect of these fractions at different concentrations (100, 200, and 400 μg/mL) was evaluated on five different cell lines (A549, HepG_2_, SH-SY5Y, HeLa, and B16). The results showed the following:

(a) For the seven SHP fractions, a decrease in proliferation was observed in all five tumor cells treated with these fractions (Figure 3). Among them, SHP-1.7 exhibited the highest growth inhibitory effect against HeLa cells, with an inhibitory rate of 43% at 200 μg/mL (Figure 3C). This inhibitory activity was the highest among all 16 fractions against HeLa cells. Similarly, at 400 μg/mL, SHP-1.0 showed the highest growth inhibitory effect against B16 cells, with the highest inhibitory rate of 43% (Figure 3B). 

(b) For the five SLP fractions, exposure to SLP led to a decrease in proliferation of all tumor cells (Figure 4). Among them, SLP-1.3 exhibited the highest anti-tumor activity among the five SLP fractions. At 100 μg/mL, SLP-1.3 showed the highest growth inhibitory effect against A549 cells, with an inhibitory rate of 38%, which was also the highest inhibitory activity of all fractions against A549 cells (Figure 4A). Similarly, at 200 μg/mL, SLP-1.3 exhibited the highest growth inhibitory effect against HepG_2_ cells, with an inhibitory rate of 24% (Figure 4D). 

(c) Among the four UPP fractions, UPP-0.5 exhibited the highest anti-tumor activity. At a concentration of 100 μg/mL, UPP-0.5 showed an inhibition rate of 19% against SH-SY5Y cells (Figure 5E). 

### 2.5. Apoptotic Effects of Polysaccharide Fractions on Tumor Cells

**Figure 6:**
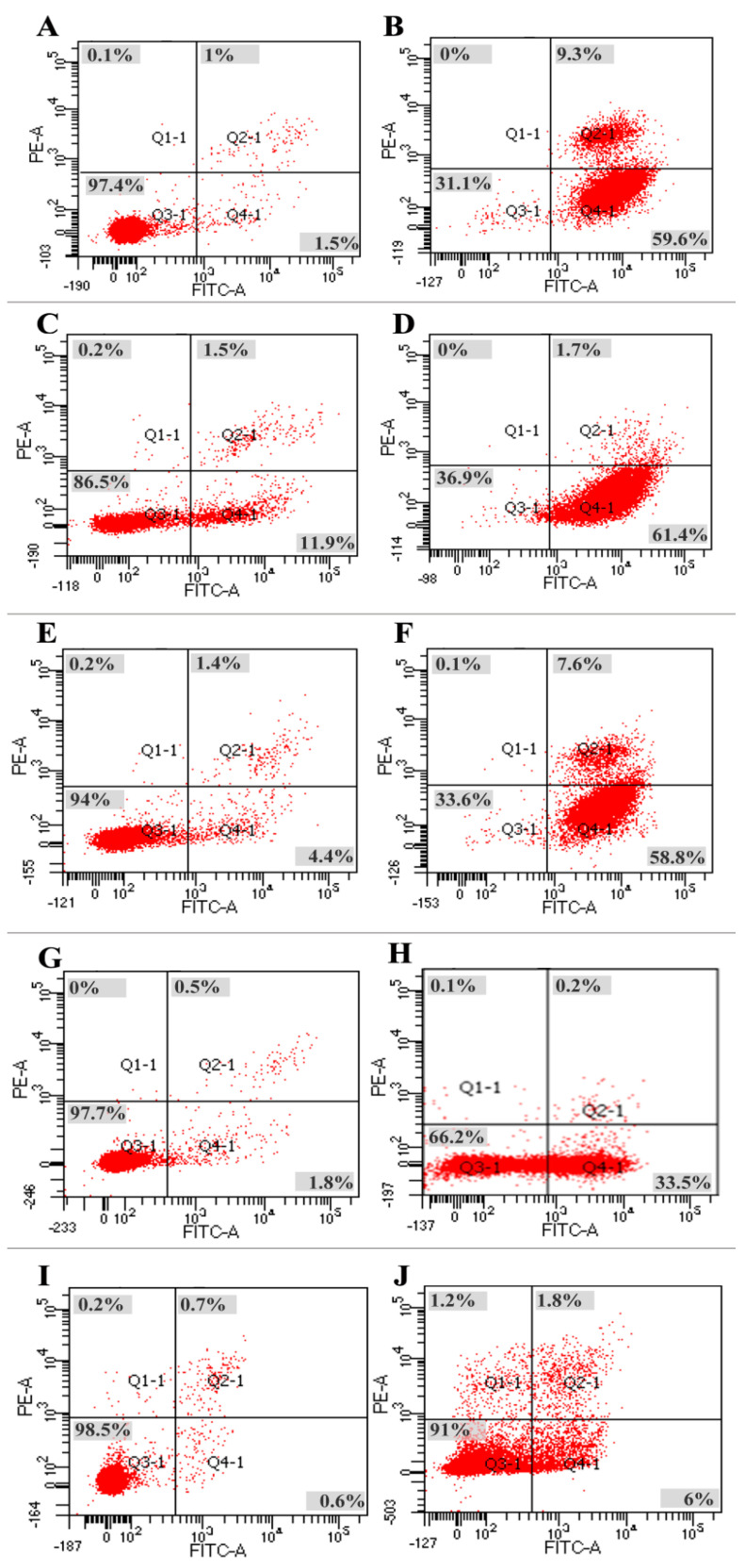
The effect of polysaccharide fractions on tumor cells through flow cytometry analysis. (**A**) Control group of HeLa cells, (**B**) 200 μg/mL SHP-1.7 treatment, (**C**) control group of B16 cells, (**D**) 400 μg/mL SHP-1.0 treatment, (**E**) control group of HepG_2_ cells, (**F**) 200 μg/mL SLP-1.3 treatment, (**G**) control group of A549 cells, (**H**) 100 μg/mL SLP-1.3 treatment, (**I**) control group of SH-SY5Y cells, and (**J**) 100 μg/mL UPP-0.5 treatment. The proportion of each area is displayed as a percentage.

To assess whether the growth inhibitory effects of polysaccharide fractions on cancer cells were associated with apoptosis, the most effective fraction–cancer cell combinations (SHP-1.7/HeLa, SHP-1.0/B16, SLP-1.3/A549, SLP-1.3/HepG_2_, and UPP-0.5/SH-SY5Y) were selected to measure their apoptosis-inducing effects. The fractions at the optimum effective concentrations (200 μg/mL SHP-1.7, 400 μg/mL SHP-1.0, 100 μg/mL SLP-1.3, 200 μg/mL SLP-1.3, and 100 μg/mL UPP-0.5) were chosen along with their corresponding tumor cells to measure their apoptosis-inducing effects. The results showed the following:

(a) For HeLa cells, after culturing with 200 μg/mL SHP-1.7 for 48 h, the apoptosis rate of HeLa cells was 68.9%, significantly higher than the apoptosis rate of 2.6% in the control group. In the experimental group, the proportion of cells in early apoptosis (Q4) was 59.6%, and the proportion of cells in late apoptosis (Q2) was 9.3% (Figure 6A,B).

(b) For B16 cells, the percentage of normal cells was 86.5% in the control group (Figure 6C,D), while in the experimental group (treated with 400 μg/mL SHP-1.0), it was 36.9%, showing a significant difference. In the experimental group, the proportion of cells in early apoptosis (Q4) was 64.1%, and the proportion of cells in late apoptosis (Q2) was 1.7%.

(c) For HepG_2_ cells, after 48 hours of incubation with SLP-1.3 (200 μg/mL), the apoptosis rate of HepG_2_ cells was 66.4%. Among them, the proportion of cells in early apoptosis (Q4) was 58.8%, and that in late apoptosis (Q2) was 7.6%. The apoptosis rate in the experimental group was significantly higher than the apoptosis rate in the control group (6.0%) (Figure 6E,F).

(d) For A549 cells, the apoptosis rate of A549 cells was 33.7% after 48 hours of incubation with 100 μg/mL SLP-1.3, significantly higher than the apoptosis rate in the control group (1.7%). Among them, the proportion of cells in early apoptosis (Q4) in the experimental group was 33.5%, and that in late apoptosis (Q2) was 0.2% (Figure 6G,H).

(e) For SH-SY5Y cells, the apoptosis rate in the control group was only 1.5%, significantly lower than the apoptosis rate of 9% in the experimental group treated with 100 μg/mL UPP-0.5 (Figure 6I,J). Among the experimental group, the proportion of cells in early apoptosis (Q4) was 6.0%, and that in late apoptosis (Q2) was 1.8%.

These data indicate that the addition of polysaccharide fractions can significantly induce apoptosis in five types of cancer cells, consistent with the results of the cancer cell proliferation assay.

### 2.6. The Effect of Polysaccharide Fractions on Lymphocyte Proliferation

**Figure 7:**
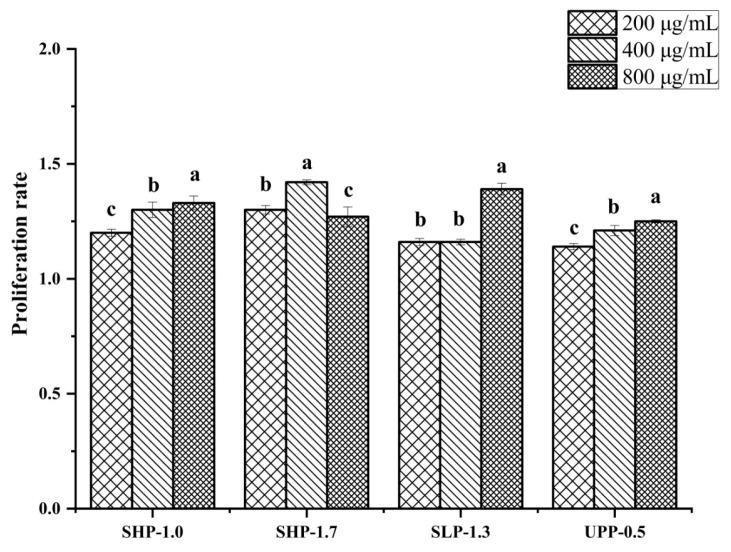
The effects of four polysaccharide fractions at different concentrations (200, 400, and 800 μg/mL) on the proliferation of macrophage (RAW264.7) cells (mean  ±  SD). The letters a–c indicate a significant difference (*p*  <  0.05) between the proliferation rate of each fraction.

The immunostimulatory activity and cytotoxicity of the four most effective fractions (SHP-1.0, SHP-1.7, SLP-1.3, and UPP-0.5) on the growth of RAW264.7 cell lines was evaluated at different concentrations (200, 400, and 800 μg/mL). The results showed that all four polysaccharide fractions could promote murine macrophage cell proliferation (Figure 7). Moreover, these polysaccharide fractions were not cytotoxic to normal cells.

### 2.7. Morphological Analysis of Apoptosis by Hoechst 33342 Staining

**Figure 8:**
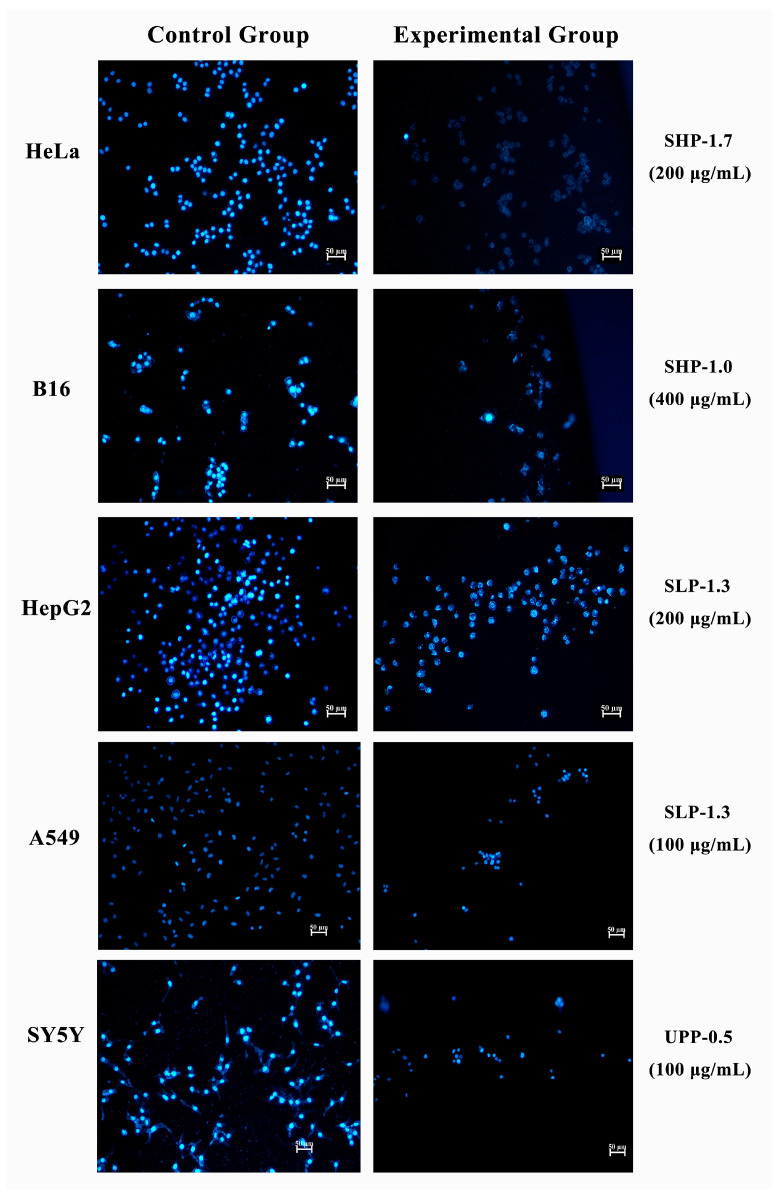
Hoechst 33342 staining in five cancer cell lines treated with the most effective fractions (SHP-1.7/HeLa, SHP-1.0/B16, SLP-1.3/A549, SLP-1.3/HepG_2_, and UPP-0.5/SH-SY5Y) for 48 h. The left side represents the control group, and the right side represents the experimental group.

To confirm the apoptotic phenotype, all five cell lines were treated with the most effective fractions (SHP-1.7/HeLa, SHP-1.0/B16, SLP-1.3/A549, SLP-1.3/HepG_2_, and UPP-0.5/SH-SY5Y). The cell-permeable DNA dye Hoechst 33342 was used to observe nuclear morphological changes. The results showed that untreated cells exhibited uniformly stained DNA with normal blue fluorescence. However, treatment with the selected fractions resulted in fragmented nuclei with condensed chromatin, which was visible as bright blue fluorescence, indicating the typical characteristics of apoptosis (Figure 8). Specifically, the SHP-1.7/HeLa, SHP-1.0/B16, and SLP-1.3/A549 combinations displayed stronger apoptotic features after staining (Figure 8). 

### 2.8. Morphological Analysis of Apoptosis by Acridine Orange Staining

**Figure 9:**
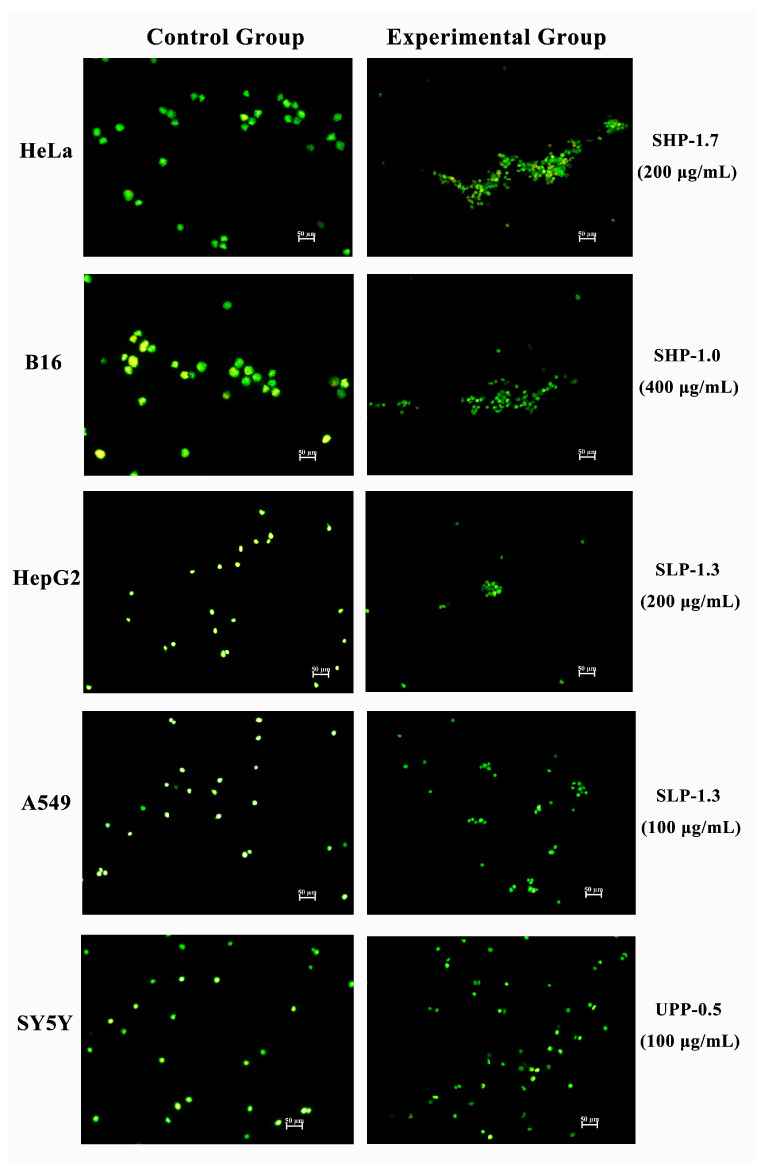
Acridine Orange (AO) staining in five cancer cell lines treated with the most effective fractions (SHP-1.7/HeLa, SHP-1.0/B16, SLP-1.3/A549, SLP-1.3/HepG_2_, and UPP-0.5/SH-SY5Y) for 48 h. The left side represents the control group, and the right side represents the experimental group.

Acridine Orange (AO) staining is another method used to detect cell apoptosis. Viable cells exhibit uniformly green nuclei, while early apoptotic cells are characterized by fragmented green patches, and late apoptotic cells display a mixture of green or orange particulate matter. In the present study, untreated cells showed green-stained unfragmented nuclei, indicating non-apoptotic live cells. However, cells treated with selected fractions exhibited highly condensed chromosomes and fragmented green nuclei, with the majority of cells showing green fragments within their nuclei and a small number of cells showing green or orange particulate matter (Figure 9). This suggests that the majority of treated cells were in the early apoptotic stage, while a few cells were in the late apoptotic stage, which is consistent with the results obtained from flow cytometry.

### 2.9. Expression of Genes Related to Tumor Apoptosis and Growth

**Figure 10:**
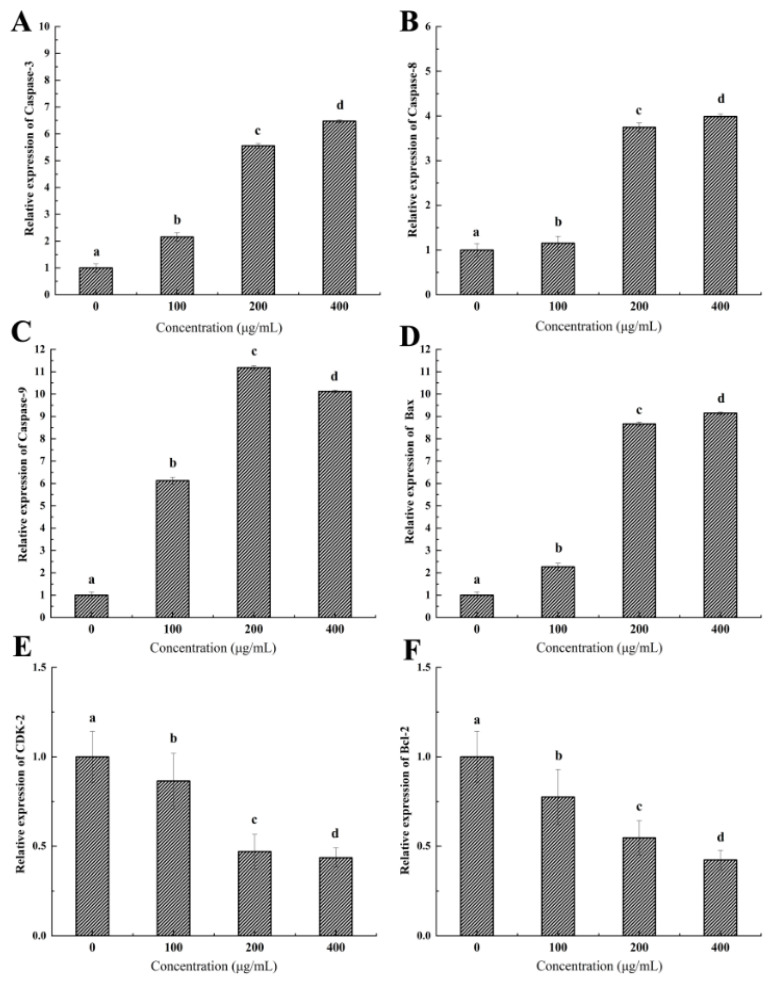
The effects of the *S. horneri* polysaccharide fraction (SHP-1.7) on the expression levels of Caspase-3, Caspase-8, Caspase-9, Bax, Bcl-2, and CDK-2 in HeLa cells. (**A**) Caspase-3, (**B**) Caspase-8, (**C**) Caspase-9, (**D**) Bax, (**E**) Bcl-2, and (**F**) CDK-2. All data are presented as mean ± SD. Significant differences (*p* < 0.05) between groups are labeled with different letters (a–d).

To investigate the potential mechanism of the anti-tumor activity of brown seaweed polysaccharides, we chose the SHP-1.7/HeLa combination as a research model to examine the expression levels of genes related to tumor apoptosis and growth, including Caspase-3, Caspase-8, Caspase-9, Bax, Bcl-2, and CDK-2, using quantitative real-time PCR (qRT-PCR). The results showed that treatment with SHP-1.7 significantly increased the expression of Caspase-3, Caspase-8, Caspase-9, and Bax in HeLa cells (Figure 10A–D). There was a concentration-dependent increase in the expression levels of Caspase-3, Caspase-8, and Bax. At a concentration of 400 μg/mL, the expression levels of Caspase-3, Caspase-8, and Bax were 6.47, 3.99, and 9.15 times higher than those of the control group, respectively. For Caspase-9, the optimal concentration was 200 μg/mL, and its expression level was 11.18 times higher than that of the control group. Additionally, SHP-1.7 treatment significantly reduced the expression of CDK-2 and Bcl-2 in HeLa cells (Figure 10E,F). There was a concentration-dependent decrease in the expression levels of CDK-2 and Bcl-2. At a concentration of 400 μg/mL, the expression levels of CDK-2 and Bcl-2 were 0.42 and 0.44 times lower than those of the control group, respectively.

### 2.10. Purification of SHP-1.0, SHP-1.7, SLP-1.3, and UPP-0.5

Based on previous biological assays, the fractions SHP-1.0, SHP-1.7, SLP-1.3, and UPP-0.5 were identified as high anti-tumor activity fractions. They were further purified using a Sephadex G-400 gel filtration column according to their molecular distribution. The results showed that after purification, the fractions SHP-1.7 and UPP-0.5 appeared to be homogeneous components, while the fractions SHP-1.0 and SLP-1.3 were mixtures that required further purification (Figure S2).

## 3. Discussion

### 3.1. Comparison of Different Anti-Tumor Activities of 16 Polysaccharide Fractions against Five Types of Tumor Cells 

With the exploration of new sources for cancer treatment drugs, one possible natural source is from seaweed [55]. Seaweed polysaccharides have been proven to be potent anti-tumor agents which exert pleiotropic and synergistic effects with chemotherapy drugs and target multiple pathways of cancer [56,57]. Increasing evidence suggests that different polysaccharides have unique anti-tumor properties against different tumor cells [6,8,17,18,19]. However, previous studies have mainly focused on analyzing individual polysaccharides or fractions, lacking systematic comparisons of the effects of different polysaccharides/fractions on different tumor cells. In this study, we selected three large brown seaweed (*S. horneri*, *S. lomentaria*, and *U. pinnatifida*), which are traditional Asian foods and have attracted much attention for their anti-tumor bioactivity. A total of 16 polysaccharide fractions were extracted from these three seaweed, with molecular weights ranging from 8.889 kDa to 729.67 kDa, and sulfate contents ranging from 0.50% to 10.77%. Additionally, the monosaccharide compositions of the isolated polysaccharide samples also varied. The types of polysaccharide fractions extracted from the three brown seaweed generally encompass those reported previously (Table 1). This provided us with a good opportunity to analyze which types of polysaccharides exhibit activity against specific tumor cells. We selected five types of aggressive tumor cells, and indeed, we discovered the specificity of polysaccharides targeting different tumor cell activities. For example, the *S. horneri* polysaccharide fraction SHP-1.7 exhibited the highest growth inhibitory effect against HeLa cells; SHP-1.0 showed the highest anti-tumor activity against B16 cells; the *S. lomentaria* polysaccharide fraction SLP-1.3 showed the highest growth inhibitory effect against A549 and HepG_2_ cells; and the *U. pinnatifida* polysaccharide fraction UPP-0.5 displayed the highest anti-tumor activity against SH-SY5Y cells. These results confirmed that different active substances have varying activities against tumor cells [8], further emphasizing the importance of developing specific anti-tumor active compounds targeting different types of tumor cells in future research. Additionally, it is necessary for future studies to conduct cross-activity comparisons of active substances under the same conditions.

### 3.2. Relationship between Anti-Tumor Activity of Brown Seaweed Polysaccharides and Their Chemical Structures 

Numerous studies have indicated a close relationship between the structural features of polysaccharides and their biological activity [54,58,59]. This diversity is attributed to their chemical structures, including molecular weight, sulfate content, monosaccharide composition, and triple-helix structure [60]. Generally, polysaccharides with a higher molecular weight exhibit stronger biological activities, as a higher molecular weight can lead to more repetitive structures binding to receptors or other membrane targets [61,62,63]. For example, in a study by Sanjeew et al., higher molecular weight sulfated polysaccharides isolated from *Sargassum horneri* were tested in vitro on RAW 264.7 cells and zebrafish models in vivo. The fraction with the highest molecular weight showed greater inhibitory activity against LPS-induced NO in RAW 264.7 macrophages [49]. Similarly, high molecular weight seaweed polysaccharides from different rea seaweed (*Chondrus armatus*, *Kappaphycus alvarezii*, and *Tichocarpus crinitus*) displayed more effective antiviral activity [64]. In our study, we found that SHP-1.0, SHP-1.7, and SLP-1.3 exhibited higher anti-tumor activity than other fractions, specifically against B16, HeLa, and A549/HepG_2_ cells, which may be attributed to their higher molecular weight (>500 kDa). There is a positive correlation between their high activity and large molecular weight in brown seaweed polysaccharides. 

Previous studies have also indicated that the anti-tumor activity of polysaccharides is closely associated with their sulfate content [65,66]. Sulfation of polysaccharides not only enhances their water solubility but also improves various biological activities such as anti-tumor, antiviral, immunostimulant, and antioxidant effects [67]. Suresh et al. suggested that the sulfate groups of polysaccharides form tight spherical conformations through intramolecular interactions and bind to cationic proteins on cell surfaces, thereby playing a crucial role in inhibiting cancer cell growth [68]. In our study, we found that SHP-1.7 exhibited potent anti-tumor activity, possibly due to its high sulfate content (10.77%).

Additionally, the different compositions of monosaccharides may also be an important reason for the variation in the anti-tumor activity of seaweed polysaccharides [69,70]. Miao et al. (2020) demonstrated that polysaccharides composed of mannose (Man) and xylose (Xyl) could improve the quality of life of tumor-bearing mice [71]. Other studies have indicated that the presence of galacturonic acid (GlcA) in the polysaccharide structure is an effective factor in enhancing its anti-tumor potential [72,73]. In the present study, 16 polysaccharide fractions exhibited their respective characteristics in the composition of monosaccharides, including the following: SHP polysaccharides are primarily composed of GlcA, followed by Man, Xyl, and fucose (Fuc), with minimal content of rhamnose (Rha), glucose (Glu), and galactose (Gal). Similarly, SLP polysaccharides are primarily composed of Man, followed by Glu, GlcA, and Rha, with lower contents of Xyl, Fuc, and Gal. Additionally, UPP polysaccharides are also primarily composed of Man, followed by Rha, Glu, and Xyl, with lower contents of Fuc, GlcA, and Gal. It is worth noting that although the overall proportion of Fuc is not high in all fractions, the Fuc content in the polysaccharide fractions with the highest anti-tumor activity is higher. SHP-1.7, SHP-1.0, and SLP-1.3 are the top three polysaccharide fractions with the highest anti-tumor activity, and also the top polysaccharide fractions with the highest Fuc content, accounting for 48.26%, 19.75%, and 11.39% of the total sugar content, respectively. This is clearly not a coincidence, but suggests a close relationship between the content of Fuc in brown algae polysaccharides and their anti-tumor activity. Similarly, studies have shown that a sulfated galactofucan from *Sargassum thunbergii*, rich in Fuc, exhibited better anti-tumor and anti-angiogenic effects on human lung cancer A549 and human umbilical vein endothelial cells, respectively [74]. 

The secondary structure of polysaccharides is another important factor influencing their anti-tumor activity [75]. The secondary structure of polysaccharide chains, formed by the glycosidic bonds linking individual monosaccharide residues, plays a crucial role in polysaccharide conformation [76]. In this study, the results of FT-IR indicated changes in the structural features of brown seaweed polysaccharides. For example, SHP and SLP showed C-H stretching vibration and α-glycosidic bonds, while UPP showed β-glycosidic bonds. Additionally, Zhang et al. demonstrated that the triple-helix conformation plays an important role in enhancing the anti-tumor effects of polysaccharides. The triple-helix sample effectively inhibited the growth of Sarcoma 180, with an inhibition ratio of 49.5%. In contrast, the bioactivity of its single chains almost disappeared [77]. In this study, all polysaccharide fractions exhibited a triple-helix structure. The differences in these structural features (secondary structure and triple-helix conformation) may be an important reason for the variation in the anti-tumor activity among the brown seaweed polysaccharides studied.

It is worth noting that the structure of seaweed polysaccharides is more complex than discussed above. There are higher-level tertiary and quaternary structures of polysaccharides, where individual units are based on the secondary structure within a complex built by non-covalent interactions (hydrogen bonds, van der Waals forces, etc.) [4,78]. In addition, spatial hindrance from acyl ester groups and electrostatic repulsion effects may alter the spatial structure of polysaccharides and the degree of sugar chain curvature, thereby increasing water solubility and leading to changes in their biological activity [79]. However, the characteristics of the complex structure of polysaccharides require a lot of work by combining various analytical methods and more precise instruments. This is a shortcoming of our study. Therefore, further structural determination of these active polysaccharides is necessary, especially those fractions that have been proven to be homogeneous components, like SHP-1.7 and UPP-0.5.

### 3.3. Apoptosis Is the Primary Reason for the Anti-Tumor Effects of Brown Seaweed Polysaccharides

Apoptosis is a physiological process of programmed cell death that plays an important role in anti-tumor treatment [13,69]. Multiple polysaccharides have been confirmed to induce apoptosis of cancer cells [80]. In our study, the flow cytometry results showed that polysaccharide fractions extracted from brown seaweed could significantly induce tumor cell apoptosis; for example, 68.9% of treated HeLa cells entered the apoptotic phase when treated with the SHP-1.7 fraction, while only 2.6% of the control group did. Additionally, most of the treated cancer cells showed ruptured incomplete nuclei and condensed chromatin through staining to observe nuclear morphology. Furthermore, cell toxicity assays confirmed the safety of brown seaweed polysaccharides. Therefore, inducing tumor cell apoptosis without harming normal cells may be the primary reason for the anti-tumor effects of brown seaweed polysaccharides.

Apoptotic factors have been shown to play an important role in regulating the anti-tumor activity of polysaccharides. A group of cysteine proteases (Caspase-3, Caspase-8, and Caspase-9) are key executors of apoptosis signaling and can induce cysteine protease-dependent apoptosis [81]. Caspase-8 and Caspase-9 control the two pathways of apoptosis, the extrinsic pathway and the intrinsic pathway, respectively [82]. In this study, qRT-PCR analysis revealed the up-regulation of Caspase-8 and Caspase-9 in HeLa cells treated with brown seaweed polysaccharides. After the initiation of apoptosis, Caspase-8 and Caspase-9 further activated the expression of the key enzyme Caspase-3 in the process of apoptosis [83], triggering a cascade reaction of the Caspase family and ultimately inducing the irreversible cell apoptotic process [84]. In this study, the expression of Caspase-3 was also significantly increased in the treated group, which further indicated the occurrence of apoptosis. 

In addition, Caspase-3 and Caspase-9 are also involved in the mitochondrial-dependent apoptotic pathway [82]. The mitochondrial-dependent apoptotic pathway is mainly regulated by the B cell leukemia/lymphoma 2 (Bcl-2) family, with more than 30 Bcl-2 members identified. Among them, Bcl-2, Bcl-x, Bcl-xL, and BAG are anti-apoptotic factors, while Bax, Bak, and Bid are pro-apoptotic factors [85]. When the proportion of pro-apoptotic factors is high, the outer mitochondrial membrane becomes perforated, releasing cytochrome C into the matrix and activating apoptotic factors such as Caspase-9 [86]. The results of this study suggest that the mitochondrial-dependent apoptotic pathway is also involved in the anti-tumor process of brown seaweed polysaccharides, as there was a significant decrease in the expression level of the anti-apoptotic factor Bcl-2 and a significant increase in the expression level of the pro-apoptotic factor, the Bax gene, in tumor cells. 

Research has also shown that the high expression of the CDK-2 gene is one of the causes of cellular carcinogenesis [87]. CDK-2 is highly expressed in various tumor cells and is considered to play an important role in the formation and development of malignant tumors [88]. Inhibiting the expression of CDK-2 can promote the apoptosis of cancer cells without harming normal cells [89]. The results of this study found that the expression of CDK-2 significantly decreased in HeLa cells treated with polysaccharides. This may be one of the reasons of brown seaweed polysaccharides’ anti-tumor activity. 

In summary, this study confirmed the mechanism by which brown seaweed polysaccharides exert anti-tumor activity at the cellular and molecular levels, which is to promote apoptosis of tumor cells. At the molecular level, brown seaweed polysaccharides can trigger a cascade reaction by increasing the ratio of Bax/Bcl-2, activating the expression of Caspase-8 and Caspase-9, and subsequently activating the expression of downstream Caspase-3, leading the cells into an irreversible death process. On the other hand, it can down-regulate the expression of Bcl-2 and CDK-2, thus inhibiting the proliferation of tumor cells.

## 4. Materials and Methods

### 4.1. Seaweed Samples

Three species of brown seaweed, *S. horneri*, *S. lomentaria*, and *U. pinnatifida*, were collected from the seas of Dalian, Yantai, and Weihai in Shandong Province, China, respectively. The authentication of the species was carried out by amplifying and sequencing a set of specific molecular markers (COI, LSU, and ITS genes). The seaweed samples were washed with distilled water, dried in a drying oven at 80 °C for 12 h, ground into powder using a grinder with a 100-mesh sieve, and then sealed in bags for use. 

### 4.2. Preparation of Crude Polysaccharides from Three Species of Brown Seaweed

We used a modified hot water extraction method to process the seaweed [54]. Briefly, the algal powder (100 g) was immersed in 30–40 times the volume (*w*/*v*) of distilled water (*S. horneri*: 30 times, *S. lomentaria*: 35 times, *U. pinnatifida*: 40 times), and the solution was refluxed at approximately 93 °C for 3–4 h (*S. horneri*: 3 h, *S. lomentaria:* 3.5 h, *U. pinnatifida*: 4 h). The supernatant was concentrated under reduced pressure and then dialyzed against distilled water for 72 h using a dialysis membrane with a molecular weight cut-off of 3500. The dialyzed fraction was precipitated by adding four times the volume of anhydrous alcohol, and then freeze-dried to obtain crude polysaccharides, which were named SHP, SLP, and UPP.

### 4.3. Purification of Polysaccharides

The crude polysaccharides were subjected to deproteinization using the Sevag method [22]. The polysaccharides were then chromatographed on a DEAE-Sepharose fast-flow column (GE Healthcare, Chicago, IL, USA). The column was eluted with a gradual gradient of NaCl concentrations: 0 M, 0.1 M, 0.3 M, 0.5 M, 0.7 M, 1 M, 1.3 M, 1.5 M, 1.7 M, and 2.0 M NaCl. The flow rate was 5.0 mL/min, with collection performed every 10 mL. The polysaccharide fractions were identified using the phenol–sulfuric acid method [90].

### 4.4. Chemical Composition Analysis 

The total carbohydrate content was determined using the phenol–sulfuric acid method with d-glucose as the standard [90]. The content of sulfate was determined using the barium chloride–gelatin method [91]. The molar ratios of monosaccharides were analyzed through derivatization with 1-phenyl-3-methyl-5-pyrazolone (PMP) [92] and measured using a high-performance liquid chromatography instrument (Shimadzu-20A, Kyoto, Japan) equipped with a YMC-Pack ODS-AQ column (250 mm × 4.6 mm, 5 μm). The performance conditions were as follows: a column temperature of 25 °C, injection volume of 100 μL (concentration of 1 mg/mL, *w*/*v*), detection wavelength of 254 nm, and mobile phase of 20% water and 80% acetonitrile. Monosaccharides (mannose, rhamnose, galacturonic acid, glucose, xylose, fucose, and galactose) from Solarbio (Beijing, China) were used as controls. Molecular weights were determined by referencing a calibration curve generated by dextran standards, with standard molecular weights of 13,050 D, 25 kD, 80 kD, 270 kD, and 670 kD. 

### 4.5. FT-IR Characterization

The FT-IR spectra of the polysaccharide samples were recorded using a Nicolet-360 FT-IR spectrometer (Thermo Fisher Scientific, Waltham, MA, USA). The scanning range was from 400 to 4000 cm^−1^ (36 scans with a resolution of 6 cm^−1^). 

### 4.6. Congo Red Analysis

A Congo red test was conducted on the polysaccharide samples with slight modifications based on the previously reported analysis method [93]. In brief, 2 mL polysaccharide solution (2.5 mg/mL) was mixed with 2 mL Congo red solution (80 μM). Subsequently, varying concentrations of a NaOH solution (final concentration of 0, 0.1, 0.2, 0.3, 0.4, and 0.5 M) were gradually added to the mixture. After incubation for 30 min at 25 °C, the absorbance of the mixture was scanned at wavelengths ranging from 200 to 600 nm. 

### 4.7. Anti-Tumor Activity Assay

#### 4.7.1. Cell Lines and Cell Culture

The human lung cancer cell line A549, human hepatoma cell line HepG_2_, and murine melanoma cell line B16 were provided by the Yellow Sea Fisheries Research Institute (Qingdao, China). The human cervical cancer cell line HeLa and human neuroblastoma cell line SH-SY5Y were provided by China University of Petroleum (East China). These cell lines were cultured in RPMI-1640 medium supplemented with 10% fetal bovine serum, 100 ng/mL streptomycin, and penicillin at 37 °C and 5% CO_2_.

#### 4.7.2. Cell Proliferation Assay

The anti-tumor activity of polysaccharide fractions on five types of cancer cells was evaluated using the Cell Counting Kit-8 (CCK-8) (Solarbio, Beijing, China) as described by Gao et al. (2021) [22]. Briefly, 50 μL of tumor cells was cultured in a 96-well cell culture plate at a concentration of 2 × 10^5^ cells/mL. A total of 50 μL of different concentrations (100, 200, and 400 μg/mL) of polysaccharide fractions was added to the cells and incubated for 24 h. Then, 100 μL of CCK-8 reagent was added to each well and incubated for an additional 0.5 h. Each well was repeated in triplicates. After incubation, the absorbance at 450 nm was measured using a microplate reader (Thermo Fisher Scientific, Waltham, MA, USA). The anti-tumor activity, expressed as the inhibition rate of cell growth, was calculated using the following formula:

Inhibition rate = 1 − AE/AC,

where AE and AC are the absorbances of the experimental and control groups, respectively.

#### 4.7.3. Flow Cytometry Analysis of Cell Apoptosis Rate

Five combinations of polysaccharide fractions with the highest anti-tumor activity and their corresponding tumor cells, including SHP-1.0 and B16 (400 μg/mL), SHP-1.7 and HeLa (200 μg/mL), SLP-1.3 and A549 (100 μg/mL), SLP-1.3 and HepG_2_ (200 μg/mL), and UPP-0.5 and SH-SY5Y (100 μg/mL), were used as the study models to determine the effect of polysaccharide addition on the apoptosis of tumor cells. In brief, these five types of tumor cells were seeded in 6-well cell culture plates (3 × 10^5^ cells/mL) and treated with the corresponding concentrations of polysaccharide fractions for 48 h. The cells were collected by centrifugation at 500 xg, washed three times with PBS, and then stained with 5 μL of annexin V-FITC and 5 μL of propidium iodide for 15 min. Subsequently, 400 μL of 1× Annexin V binding buffer (TransGen Biotech, Beijing, China) was added and mixed, and the apoptosis rate of the cells was determined within 1 h using an FACS Aria II flow cytometer (Becton Dickinson, Franklin Lakes, NJ, USA).

#### 4.7.4. RAW264.7 Cell Proliferation/Cytotoxicity Assay

RAW264.7 macrophages were provided by the Yellow Sea Fisheries Research Institute and cultured in DMEM medium in a 96-well cell incubator (37 °C, 5% CO_2_) at a density of 5 × 10^5^ cells/mL. Cells were treated with solutions of each of the four fractions (SHP-1.0, SHP-1.7, SLP-1.3, and UPP-0.5) at concentrations of 200, 400, and 800 μg/mL for 24 h. The control group was incubated with PBS instead of the polysaccharide solution. A total of 20 μL of CCK-8 solution was added to each well and incubation continued for 4 h. The absorbance was measured at 450 nm using a microplate reader. The proliferation rate was calculated using the following formula:

Proliferation rate (%) = AE/AC × 100%,

where AE and AC are the absorbance values of the experimental and control groups, respectively.

#### 4.7.5. Hoechst 33342 Staining Assay

The five types of tumor cells were cultured in 96-well plates at a density of 2 × 10^5^ cells/mL. Cells were treated with corresponding concentrations of polysaccharide fractions for 48 h. Subsequently, 100 μL of Hoechst 33342 staining solution was added to each well, and incubation was continued for 0.5 h. The cells were then washed twice with PBS and imaged under a fluorescence microscope (Nikon Eclipse 80i microscope, Tokyo, Japan).

#### 4.7.6. AO Staining Assay

The procedure for the AO staining assay is similar to the Hoechst 33342 staining assay with slight modifications. The five types of tumor cells (2 × 10^5^ cells/mL) were treated with corresponding concentrations of polysaccharide fractions for 48 h. Subsequently, 100 μL of AO staining solution was added to each well, and incubation was continued for 15 min. The cells were washed twice with PBS and imaged under a fluorescence microscope.

#### 4.7.7. Quantitative Real-Time PCR (qRT-PCR) Analysis

HeLa cells were cultured in 6-well culture plates at a cell density of 3 × 10^5^ cells/mL. The experimental group was treated with 200 μL/mL of SHP-1.7, while the control group was treated with PBS as a substitute. After 48 h of treatment, total RNA was extracted from the cells using Trizol reagent (Thermo Fisher Scientific, Waltham, MA, USA) and transcribed into cDNA using a Reverse Transcription System kit (Takara, Beijing, China) following the manufacturer’s instructions. qRT-PCR was performed with the incorporation of SYBR Green (Takara, Beijing, China) using Rotor-Gene Q (Qiagen, Hilden, Germany). The expression levels of the genes Caspase-3, Caspase-8, Caspase-9, CDK-2, Bax, and Bcl-2 were calculated using the comparative CT method [94]. β-Actin was used as an internal reference gene. The primer sequences used are listed in Table 5.

### 4.8. Purification of the High Anti-Tumor Activity Fractions

The fractions SHP-1.0, SHP-1.7, SLP-1.3, and UPP-0.5 were selected for further purification using a Sephadex G-400 gel-filtration column (4 cm × 80 cm) (Solarbio, Beijing, China) according to their molecular distribution. Distilled water was used as the eluent at a flow rate of 0.5 mL/min. A phenol–sulfuric acid assay was used to identify the purified fractions.

### 4.9. Statistical Analyses

All experiments were repeated in triplicate and analyzed using SPSS 19.0 software (SPSS Inc., Chicago, IL, USA). The results are presented as mean ± standard deviation (SD). Significant differences (*p* < 0.05) between the values were analyzed using one-way analysis of variance and tested with Duncan’s test. 

## 5. Conclusions

In this study, 16 polysaccharide fractions with different chemical compositions and structures were extracted and purified from three large brown seaweed (*S. horneri*, *S. lomentaria*, and *U. pinnatifida*). This provided us with a good opportunity to compare the anti-tumor activities of so many polysaccharides/fractions under the same conditions. We selected five tumor cells for anti-tumor activity testing, representing five severe tumors, and the results further demonstrated the uniqueness of polysaccharide anti-tumor properties. The results showed that a specific polysaccharide was not consistently the most effective for all tumor cells but rather different polysaccharide fractions exhibit varying activities against different tumor cells. This diversity in anti-tumor properties may be attributed to the varying chemical compositions and structures of polysaccharides, such as the molecular weight, sulfate content, monosaccharide composition, and secondary structure of polysaccharides. In addition, inducing apoptosis in tumor cells appears to be a primary mechanism behind the anti-tumor effects of brown seaweed polysaccharides, involving factors such as Caspases, Bax, Bcl-2, and CDK-2. This study provides support for obtaining highly efficient anti-tumor activity fractions from brown seaweed polysaccharides and suggests the necessity to design specific anti-tumor activity fractions tailored to different types of tumor cells in future research, aiming for enhanced therapeutic outcomes. 

## Figures and Tables

**Table 1:** Previous studies on the three seaweed polysaccharide fractions and their biological activity analysis.

Species	Polysaccharide Fractions	Molecular Weight	Studied Activity	**References**
*Sargassum horneri*	SHS1 and SHS0.5	--	Antioxidant activity	[31]
	SHP30, SHP60, and SHP80	1.58 × 10^3^ kDa, 1.92 × 10^3^ kDa, and 11.2 kDa	Antioxidant and anti-tumor (gastric cancer and DLD intestinal cancer) activity	[29]
	SHPSA	5.78 × 10^5^ kDa	Anti-tumor (DLD intestinal cancer) activity	[28]
	SHPH1 and SHPH2	78.31 kDa and 21.42 kDa	Antioxidant and moisture-preserving activities	[32]
	F1 and F2	--	Anti-inflammatory activity	[33]
	F1, F2, F3, and F4	<5 kDa, 5–10 kDa, 10–30 kDa, and >30 kDa	Anti-inflammatory activity	[34]
	SHCF1 and SHCF2	>50 kDa and <50 kDa	Anti-inflammatory activity	[35]
	F1, F2, and F3	63 kDa, 114 kDa, and 138 kDa	Anti-tumor (human colon carcinoma cancer) and radiosensitizing activities	[23]
*Scytosiphon lomentaria*	A5, A10, A20, A30, and A40	11.2 kDa,10.6 kDa,13.3 kDa, 8.5 kDa, and 12.1 kDa	Antiviral activity	[36]
	SLCF1, SLCF2, SLCF3, SLCF4, and SLCF5	--	Anti-inflammatory activity	[37]
*Undaria pinnatifida*	PSU1-4	--	Anti-inflammatory and antioxidant activities	[38]
	SPUP	97.9 kDa	Anti-tumor (breast cancer and ovarian cancer) activity Anti-atherosclerotic effects	[39,40,41,42]
	UPPS-B1 and S-UPPS-B1	37 kDa and 110 kDa	Anti-tumor (ascites tumor) activity	[43]
	Up-3, Up-4, and Up-5	84.8 kDa, 41.4 kDa, and 330.7 kDa	Hypoglycemic activity	[44]
	UPP 1, UPP 2, and UPP 3	7.212 kDa, 13.924 kDa, and 55.875 kDa	Immunostimulatory activity	[45]
	UPE_8, UPE_8S, and UPE_8P	1062kDa, 447 kDa, and 745 kDa	Antioxidant activity	[46]

**Table 2:** Chemical composition of *S. horneri* polysaccharide (SHP) fractions.

Fractions	Total Saccharides	Sulfate Contents	Molecular Weight (kDa)	Monosaccharide Composition (Molar Ratio)						
				Man	Rha	GlcA	Glu	Xyl	Fuc	Gal
SHP-0.3	32%	7.62%	10.493	0.468	1.1	Nd	0.126	9.18	Nd	Nd
SHP-0.5	26%	6.49%	30.945	0.725	Nd	2.29	Nd	0.771	Nd	Nd
SHP-0.7	19%	6.91%	82.341	2.2	1.44	1.26	1.65	1.76	0.328	Nd
SHP-1.0	36%	2.2%	614.40	5.22	3.377	Nd	0.497	Nd	2.256	0.07
SHP-1.3	18%	2.91%	620.64	2.46	Nd	19.9	0.37	1.82	3	0.17
SHP-1.5	23%	8.63%	627.85	7.18	Nd	1.67	0.21	1.19	2.32	1.38
SHP-1.7	37%	10.77%	729.67	Nd	Nd	2.46	1.44	0.85	4.43	Nd

Man: mannose; Rha: rhamnose; GlcA: galacturonic acid; Glu: glucose; Xyl: xylose; Fuc: fucose; Gal: galactose; Nd: not detected.

**Table 3:** Chemical composition of *S. lomentaria* polysaccharide (SLP) fractions.

Fractions	Total Saccharides	Sulfate Contents	Molecular Weight (kDa)	Monosaccharide Composition (Molar Ratio)						
				Man	Rha	GlcA	Glu	Xyl	Fuc	Gal
SLP-0.3	24%	2.34%	301.31	4.347	0.076	0.05	0.842	0.021	0.333	0.01
SLP-0.5	30%	2.06%	375.76	0.0538	0.215	Nd	0.0323	0.2	0.016	0.027
SLP-0.7	75%	6.49%	448.64	0.154	0.832	3.921	1.218	0.068	0.069	0.086
SLP-1.3	60%	2.48%	518.36	0.96	0.182	0.813	0.424	Nd	0.317	0.087
SLP-1.5	36%	1.49%	646.83	0.091	0.0935	Nd	0.836	Nd	0.0347	0.075

Man: mannose; Rha: rhamnose; GlcA: galacturonic acid; Glu: glucose; Xyl: xylose; Fuc: fucose; Gal: galactose; Nd: not detected.

**Table 4:** Chemical composition of *U. pinnatifida* polysaccharide (UPP) fractions.

Fractions	Total Saccharides	Sulfate Contents	Molecular Weight (kDa)	Monosaccharide Composition (Molar Ratio)						
				Man	Rha	GlcA	Glu	Xyl	Fuc	Gal
UPP-0.3	90%	0.50%	8.889	4.302	Nd	Nd	1.032	Nd	0.418	Nd
UPP-0.5	13%	1.05%	341.01	0.596	Nd	Nd	1.002	Nd	0.011	Nd
UPP-1.0	42%	1.34%	402.35	Nd	6.29	Nd	0.105	Nd	0.942	0.533
UPP-1.3	25%	1.06%	444.96	0.974	Nd	1.22	0.966	7.05	0.441	Nd

Man: mannose; Rha: rhamnose; GlcA: galacturonic acid; Glu: glucose; Xyl: xylose; Fuc: fucose; Gal: galactose; Nd: not detected.

**Table 5:** The list of primers used in this study.

Gene	Primer	Sequence (5′–3′)
Caspase 3	F R	TGACATCTCGGTCTGGTA AACATCACGCATCAATTCC
Caspase 8	F R	TTCCTGAGCCTGGACTACATT GAAGTTCCCTTTCCATCTCCT
Caspase 9	F R	ACTAACAGGCAAGCAGCAAA CCAAATCCTCCAGAACCAAT
CDK 2	F R	AACACAGAGGGGGCCATCAAGC CAGGAGCTCGGTACCACAGGGTC
Bax	F R	AGGTCTTTTTCCGAGTGGCAGC CCCGGAGGAAGTCCAATGTCC
Bcl-2	F R	ATGTGTGTGGAGAGCGTCAA GAGACAGCCAGGAGAAATCA
β-actin	F R	CGTGGACATCCGCAAAG GCACTCGTCATACTCCTGCTT

## Data Availability

Data is contained within the article.

## Supplementary Materials

The following supporting information can be downloaded at: https://www.mdpi.com/article/10.3390/md22070316/s1, Figure S1: DEAE-sepharose Fast Flow chromatographic profiles of polysaccharide fractions from three types of seaweed; Figure S2: The elution curve of (**A**) SHP-1.0, (**B**) SHP-1.7, (**C**) SLP-1.3, and (D) UPP-0.5 on the Sephadex G-400 gel chromatography column.
