# Supplementary material for: A Comparative Analysis of the Anti-Tumor Activity of Sixteen Polysaccharide Fractions from Three Large Brown Seaweed, Sargassum horneri, Scytosiphon lomentaria, and Undaria pinnatifida
Source: Mar Drugs. 2024 Jul 16;22(7):316. doi: 10.3390/md22070316 (PMC11278018; doi:10.3390/md22070316)
Supplement: Supplementary file 1 [file marinedrugs-22-00316-s001.zip › Figure S.pdf]

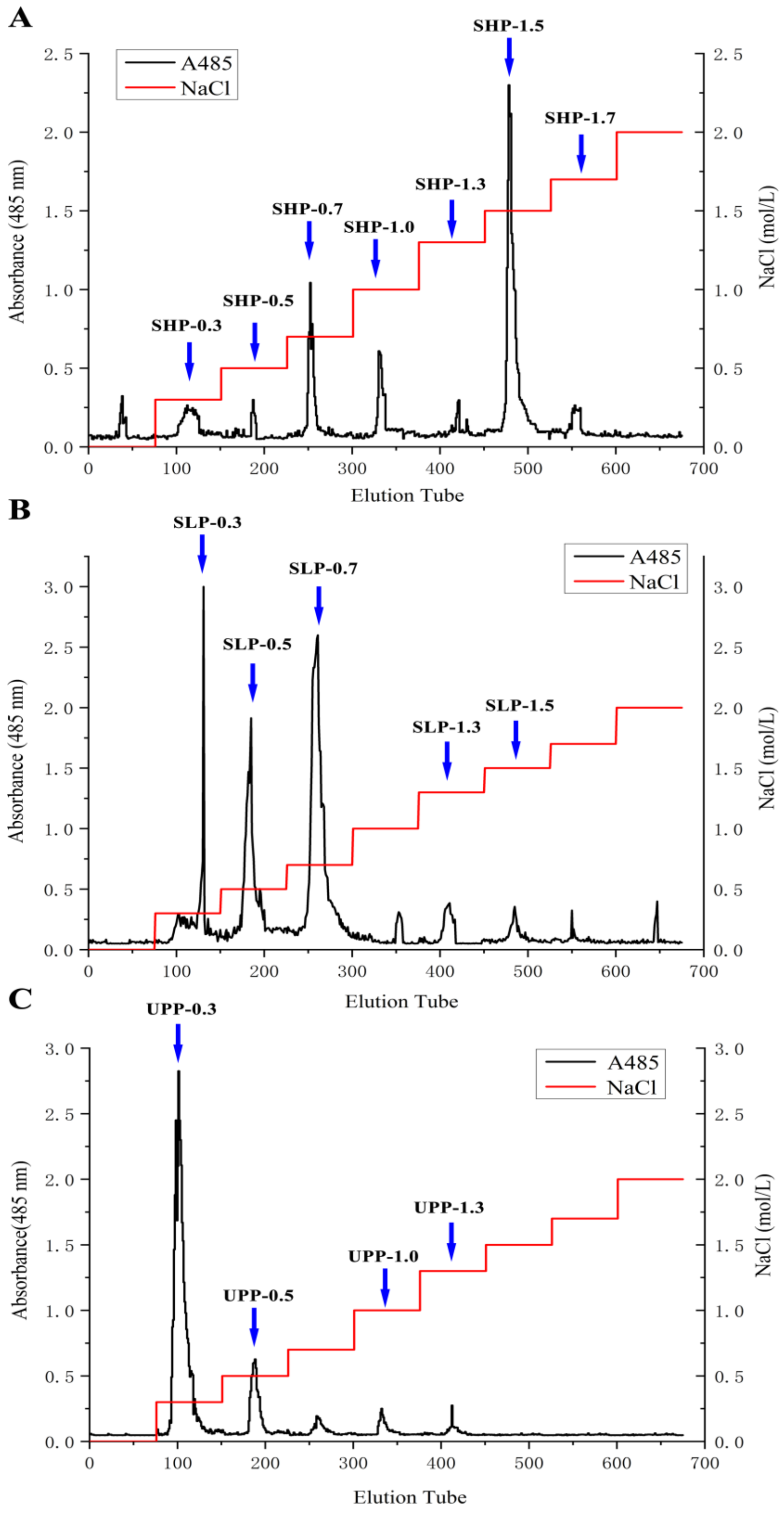

Figure S1. DEAE-sepharose Fast Flow chromatographic profiles of polysaccharide fractions from three types of seaweed. (A) *Sargassum horneri* polysaccharide (SHP) fractions, (B) *Scytosiphon lomentaria* polysaccharide (SLP) fractions, (C) *Undaria pinnatifida* polysaccharide (UPP) fractions. The collected fractions are indicated by blue arrows.

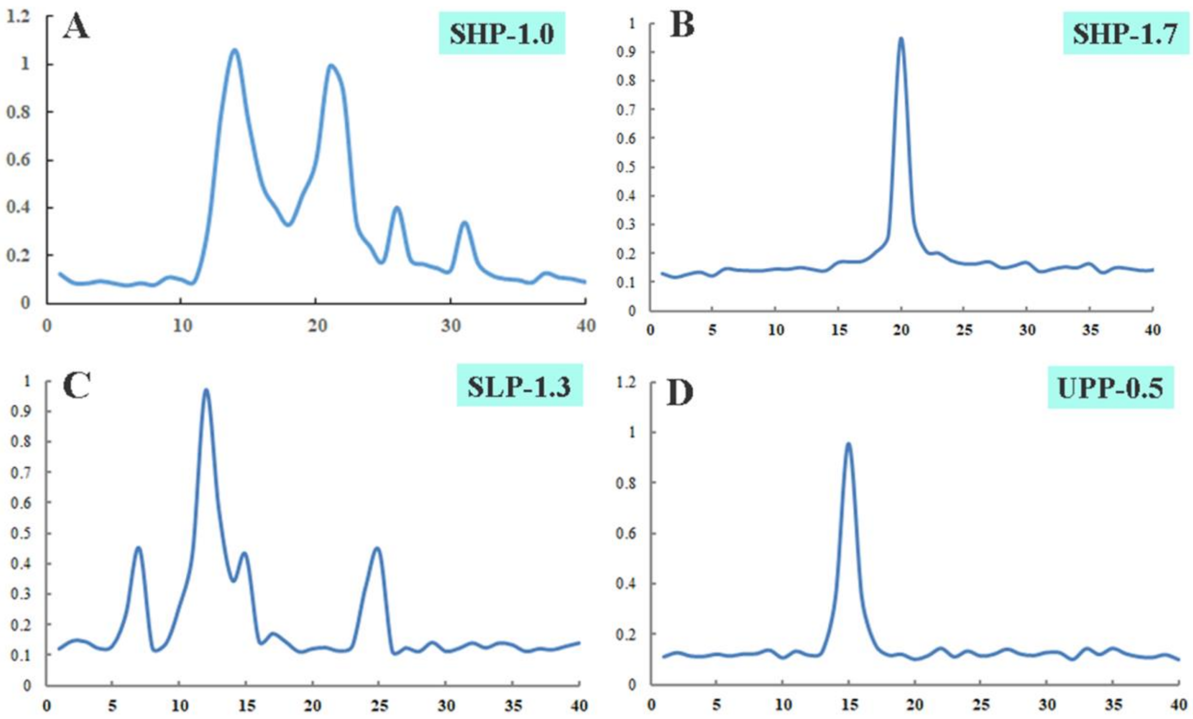

Figure S2. The elution curve of (A) SHP-1.0, (B) SHP-1.7, (C) SLP-1.3, and (D) UPP-0.5 on the Sephadex G-400 gel chromatography column.
